# Resisting the resistance: the antimicrobial peptide DGL13K selects for small colony variants of *Staphylococcus aureus* that show increased resistance to its stereoisomer LGL13K, but not to DGL13K

**DOI:** 10.1128/jb.00505-24

**Published:** 2025-06-04

**Authors:** Sven-Ulrik Gorr

**Affiliations:** 1 Department of Diagnostic and Biological Science, University of Minnesota 5635, Minneapolis, Minnesota, USA; University of Illinois Chicago, Chicago, Illinois, USA

**Keywords:** antimicrobial peptides, *Staphylococcus aureus*, small colony variants, stereoisomer, menaquinone, bacterial resistance

## Abstract

About 30% of the population are nasal carriers of *Staphylococcus aureus*, a leading cause of bacteremia, endocarditis, osteomyelitis, and skin and soft tissue infections. Antibiotic-resistant bacteria, in particular, are an increasing problem in both hospital and community settings. In this study, we sought to determine the cellular consequences of long-term exposure of *S. aureus* to the antimicrobial peptide stereoisomers, DGL13K and LGL13K. Both peptides selected for mutations in the chorismate/menaquinone biosynthetic pathway, which resulted in increased resistance to LGL13K but not DGL13K. DGL13K-selected isolates showed a mutation in *aroF*, while *menA* and *menH* were mutated in LGL13K-selected isolates. The latter also contained a mutation of *frsA*. The peptide-selected isolates exhibited golden coloration, suggesting increased production of the carotenoid staphyloxanthin, which could enhance resistance to cationic antimicrobial peptides (AMPs). The peptide-selected isolates grew as small colony variants, which have also been associated with resistance to AMPs. Addition of menaquinone to the growth medium reduced the generation time of DGL13K-selected mutants, but not LGL13K-selected mutants. Instead, the latter showed an increased MIC to LGL13K and greatly reduced ATP levels. The peptide-selected isolates showed increased biofilm formation and decreased autolysis, which was further reduced by alkaline shock, consistent with increased Asp23 expression. The mechanisms behind the differential effect of DGL13K and LGL13K on *S. aureus* resistance remain to be elucidated. The finding that DGL13K induced resistance to the stereoisomer LGL13K but not to DGL13K itself suggests that peptide primary structure is responsible for inducing bacterial defense mechanisms, but the peptide secondary structure determines if the defense mechanisms are effective against each peptide.

**IMPORTANCE:**

This work examines resistance to stereoisomers of the antimicrobial peptide GL13K in *Staphylococcus aureus*. Both DGL13K and LGL13K isomers cause mutations in the menaquinone pathway. While LGL13K causes resistance to LGL13K, the bacteria remain susceptible to DGL13K. Conversely, DGL13K also raises resistance to LGL13K, but the cells remain susceptible to DGL13K. The resistant isolates exhibit a small colony variant phenotype and overproduce the pigment staphyloxanthin. Menaquinone supplementation decreases the long generation time of DGL13K-selected isolates and increases the MIC of LGL13K-selected isolates.

## INTRODUCTION

Mucosal surfaces are exposed to both commensal and invading bacteria, which must survive in an environment rich in host-defense molecules, including antimicrobial peptides (AMPs) (1). As an example, about 30% of the population are nasal carriers of *Staphylococcus aureus*, which has been associated with systemic infections, including bacteremia (2). Although decolonization efforts, including a nasal antiseptic, can reduce hospital-acquired methicillin-resistant *S. aureus* (MRSA) rates, it may come with a cost of increased antiseptic resistance (3). A highly adaptable metabolism allows *S. aureus* to infect various host environments (4), and it is a leading cause of bacteremia, endocarditis, osteomyelitis, skin, and soft tissue infections. Antibiotic-resistant bacteria, in particular, are an increasing problem in both hospital and community settings. As an example, the U.S. Centers for Disease Control and Prevention estimates that over 300,000 infections and 10,000 deaths annually are attributable to MRSA, which is listed as a serious threat by the agency (5). WHO also lists *S. aureus* as a high-priority pathogen for which novel antibiotics are urgently needed (6).

A dozen cationic AMPs mediate the antibacterial function of human nasal fluid (7), and 45 AMPs have been identified in the oral cavity (8). Bacteria use a variety of mechanisms to defend against AMPs, including electrostatic repulsion, cell wall alteration, membrane alteration, proteolysis, protein binding, and efflux pumps (9–12). Accordingly, a number of resistance genes have been identified in Gram-positive bacteria (13), including two dozen resistance genes—the “resistome,” which are associated with resistance to the AMP LL-37 (14). The identification of diverse resistance genes, belonging to different functional families, points to the plasticity of bacterial resistance. As an example, the Gram-positive bacteria *Enterococcus faecalis* and *Streptococcus gordonii* are resistant to the AMP LGL13K. However, deletion of *dltA*, which functions in D-alanylation of teichoic acids, renders the bacteria sensitive to LGL13K. Upon prolonged exposure to LGL13K, these *dltA^−^* bacteria develop *de novo* resistance to this peptide through a separate mechanism (15).

Host-defense peptides have served as inspiration for the design of therapeutic AMPs (9, 16). To be successful, a therapeutic AMP must overcome multiple resistance mechanisms, which has raised the concern that resistance to therapeutic AMPs could also render bacteria resistant to host-defense peptides (“arming the enemy”) (17, 18). Indeed, cross-resistance has been achieved experimentally *in vitro* and in animal models (19–21), but this is not a consistent outcome of AMP selection, which may increase, decrease, or have no effect on the minimum inhibitory concentration (MIC) of a different antimicrobial peptide; reviewed in reference 9. Moreover, the widespread use of nisin and polymyxin B, without generalized resistance, suggests that this is not a general threat to host defenses (22).

We designed the AMP LGL13K, which kills Gram-negative bacteria and their biofilms (23, 24). However, LGL13K is not effective against Gram-positive bacteria and is susceptible to proteolysis. To overcome this problem, an all-D-amino acid isomer, DGL13K, which resists proteolysis, was designed (23). DGL13K is highly effective against Gram-positive bacteria, and this activity is independent of proteolytic activity (15). In this report, we extend these studies to *S. aureus* and show that these Gram-positive bacteria are also resistant to LGL13K but not DGL13K. Remarkably, either peptide isomer increases resistance to LGL13K but not DGL13K. The resistant bacteria contain mutations in the shikimate/menaquinone biosynthetic pathways and exhibit a small colony variant (SCV) phenotype that differs from traditional SCVs.

## MATERIALS AND METHODS

### Bacterial cultures

*Pseudomonas aeruginosa* Xen41, a bioluminescent derivative of *P. aeruginosa* PAO1, was obtained from Xenogen (Alameda, CA; now Revvity, Waltham, MA). *Staphylococcus aureus* Xen36, a bioluminescent derivative of American Type Culture Collection strain 49525, was obtained from Revvity. *P. aeruginosa* and *S. aureus* were cultured from glycerol stocks at 37°C with constant shaking at 200 rpm in liquid cultures prepared in Luria-Bertani broth and Todd Hewitt Broth (THB) (Difco, Franklin Lakes, NJ), respectively. Overnight cultures typically reached an optical density at 600 nm (OD600) corresponding to 5 × 10^8^ CFU/mL for *P. aeruginosa* and 3 × 10^8^ CFU/mL for *S. aureus*. Colonies of the latter were isolated on 1.5% agar prepared in THB and cultured at 37°C.

### Peptides

**Fig 1:**
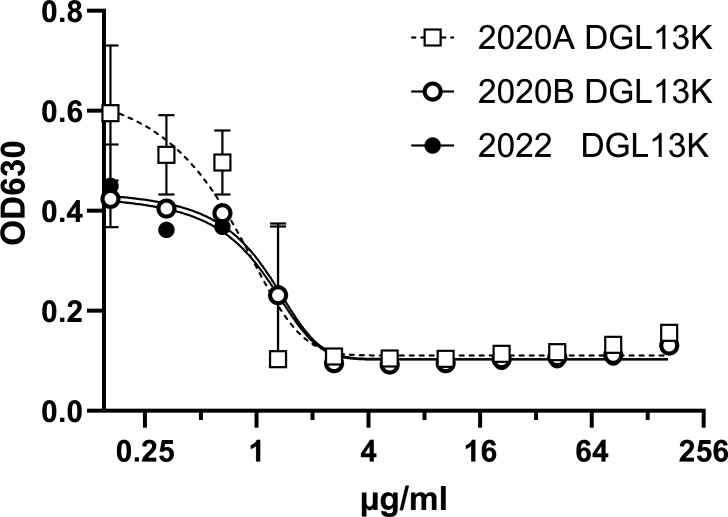
DGL13K retains activity in solution. Two stock solutions of DGL13K were stored at 4°C for 2 years (2020 A □ and B ○) or freshly made (2022 ●). MIC was tested against *S. aureus* Xen 36. Data are shown as mean ± range of duplicate samples, *N* = 2.

LGL13K and DGL13K were purchased from AappTec (Louisville, KY) or Bachem (Torrance, CA) at >95% purity. Peptide identity and purity were confirmed by the supplier by mass spectrometry and reversed-phase high-performance liquid chromatography, respectively. Peptides were dissolved at 10 mg/mL in sterile 0.01% acetic acid, and these stock solutions were stored at 4°C until use. Peptide batches were tested for antimicrobial activity by MIC assays. The stock solutions retained activity for at least 2 years at 4°C (Fig. 1).

### Minimum inhibitory concentration

MIC assays were performed essentially as previously described (15, 25). The overnight cultures were diluted to about 10^5^ CFU/mL in THB for *S. aureus* and Mueller-Hinton Broth (Difco) for *P. aeruginosa*. Diluted bacteria (100 µL/well) were incubated in 96-well polypropylene microtiter plates with twofold serial dilutions of each peptide in 20 µL “10% PBS” (a 10-fold dilution of PBS in sterile water). LGL13K was tested in the concentration range 1,667 µg/mL to 1.6 µg/mL, while DGL13K was tested in the range 167 µg/mL to 0.16 µg/mL. Control samples without added peptide were included in each assay. Culture plates were incubated overnight at 37°C with constant shaking on a Stovall Belly Dancer lab shaker at speed 5 (IBI, Dubuque, IA). The OD630 was determined in a BioTek Synergy HT plate reader (BioTek, Winooski, VT; now Agilent, Santa Clara, CA). The OD630 for each peptide dilution was plotted, and the MIC was determined in four parallel replicates. In some experiments, the MIC was determined in the presence of 50 µM menaquinone (Vitamin K2; Supelco), which was added from a 5 mM stock solution in 95% ethanol.

For serial MIC assays (25), a single representative well, containing a peptide concentration twofold lower than the MIC (0.5× MIC), was sampled, and the bacteria were diluted 1,100× in THB or Mueller-Hinton Broth and used to inoculate the next MIC assay. Sampling in two separate experiments was repeated for 6 days for *S. aureus* and 12 days for *P. aeruginosa*.

### Peptide-selected isolates

Aliquots of *S. aureus* cultures, which were treated with DGL13K or LGL13K in 5–6 consecutive MIC assays, were streaked on THB agar or cultured overnight in the absence of DGL13K or LGL13K and then streaked on THB agar. Individual colonies were selected and expanded in THB, in the absence of DGL13K or LGL13K, overnight at 37°C with shaking at 200 rpm. The overnight cultures of individual colonies were mixed with glycerol (10% final concentration) and stored at −80°C as *DGL13K-selected and LGL13K-selected isolates,* respectively.

### Genome sequencing

Bacterial pellets of peptide-selected isolates were submitted to the University of Minnesota Genomics Center for DNA purification, genomic library generation, and MiSeq genomic sequencing. gDNA samples were converted to Illumina sequencing libraries using Illumina’s DNA Prep Sample Preparation Kit (Illumina, San Diego, CA). Briefly, 1–500 ng of gDNA was simultaneously fragmented and tagged with a unique adapter sequence. The DNA was simultaneously indexed and amplified by PCR. Final library size distribution was validated using capillary electrophoresis and quantified using PicoGreen fluorimetry.

Libraries were sequenced on an Illumina MiSeq platform (2 × 300 bp) using Illumina’s SBS chemistry. Primary analysis and de-multiplexing were performed using Illumina’s bcl-convert v4.0.3. The resulting FASTQ files were mapped to the published *S. aureus* reference genome sequence (NCBI reference sequence: NC_002950.2) via BWA (0.7.17-r1188) to generate BAM files. Variant calling was done in parallel across all samples via Freebayes using a minimum variant frequency of 0.01 and a minimum coverage of 34 reads. Polymorphism frequencies in each culture were determined and gated at a >10% threshold. Raw data files associated with genome sequencing are maintained by the Minnesota Supercomputing Institute (https://www.msi.umn.edu/).

### Growth curves and colony size

Aliquots (5 µL) of glycerol stocks of peptide-selected isolates were diluted in 1 mL THB, and 200 µL aliquots were cultured in 96-well plates at 37°C without shaking. OD630 was read at 45–90 min intervals and fitted to an exponential growth curve, which was used to calculate generation time (doubling time) (Graphpad Prism 8; GraphPad Software, Boston, MA).

To visualize differences in colony size, overnight cultures of wild-type and peptide-selected isolates were diluted in THB, and an aliquot was spread on THB agar and again cultured overnight at 37°C.

### Colony pigmentation

To determine colony pigmentations, aliquots of wild-type or peptide-selected isolates were plated on blood agar containing 50 µg/L menadione, 5 mg/L hemin, and 0.25% sheep blood. The plates were incubated for 2 days at 37°C.

### Menaquinone supplementation

To determine if menaquinone supplementation affected growth rate, wild-type and peptide-selected isolates were cultured in Mueller-Hinton broth alone or supplemented with 50 µM menaquinone (Vitamin K2; Supelco), which was added from a 5 mM stock solution in 95% ethanol. Exponential growth curves were determined, and the generation time was calculated, as described above.

### Cellular ATP content

*S. aureus* Xen 36 contains a stable copy of the modified *Photorhabdus luminescens* luxABCDE operon, which utilizes cellular ATP to emit light. We employed this system to determine cellular ATP content, as a measure of metabolic activity (26). Bacteria were cultured to the stationary phase, and the bacterial luminescence (relative light units—RLU) was recorded and expressed relative to OD600 of the culture, to account for different growth rates.

### Biofilm analysis

Bacteria were cultured in 200 µL THB/well in a 96-well polystyrene plate for 48 h at 37°C with gentle rocking. The OD630 was measured at the end of the culture period to determine culture density.

Unattached bacteria were aspirated, and adhered bacteria were washed with 300 µL PBS and then stained with 0.03% crystal violet for 30 min at room temperature. The wells were washed with 2 × 300 µL PBS, followed by incubation with 95% ethanol for 30 min at 37°C to dissolve biofilm-associated crystal violet. The dissolved crystal violet solutions from four wells were combined, and OD570 was determined (27). The reading for each sample was normalized to the culture density to account for differences in growth rates between different strains.

### Alkaline shock

Overnight cultures of each strain were diluted 10-fold in THB and cultured to log phase (OD630 = 0.5–0.9). The bacteria were pelleted and resuspended in THB adjusted to pH 7 or pH 10 and incubated for 1 h at 37°C before use in the autolysis assay.

### Autolysis assay

Autolysis was performed as previously described with minor modifications (15). Bacteria that had been pre-incubated at pH 7 or pH 10 (alkaline shock) were pelleted and resuspended in PBS and then pelleted and resuspended in ice-cold dH_2_O. The bacteria were again pelleted and then resuspended in PBS with 0.05% Triton X-100, as previously described (15). The OD630 was monitored spectrophotometrically for 3 h. Percent autolysis was calculated from the absorbance at T = 0 h and T = 2 h: [1 − (OD630_T=0_ – OD630_T=2_)/OD630_T=0_] × 100%.

## RESULTS

### Resistance to LGL13K or DGL13K

**Fig 2:**
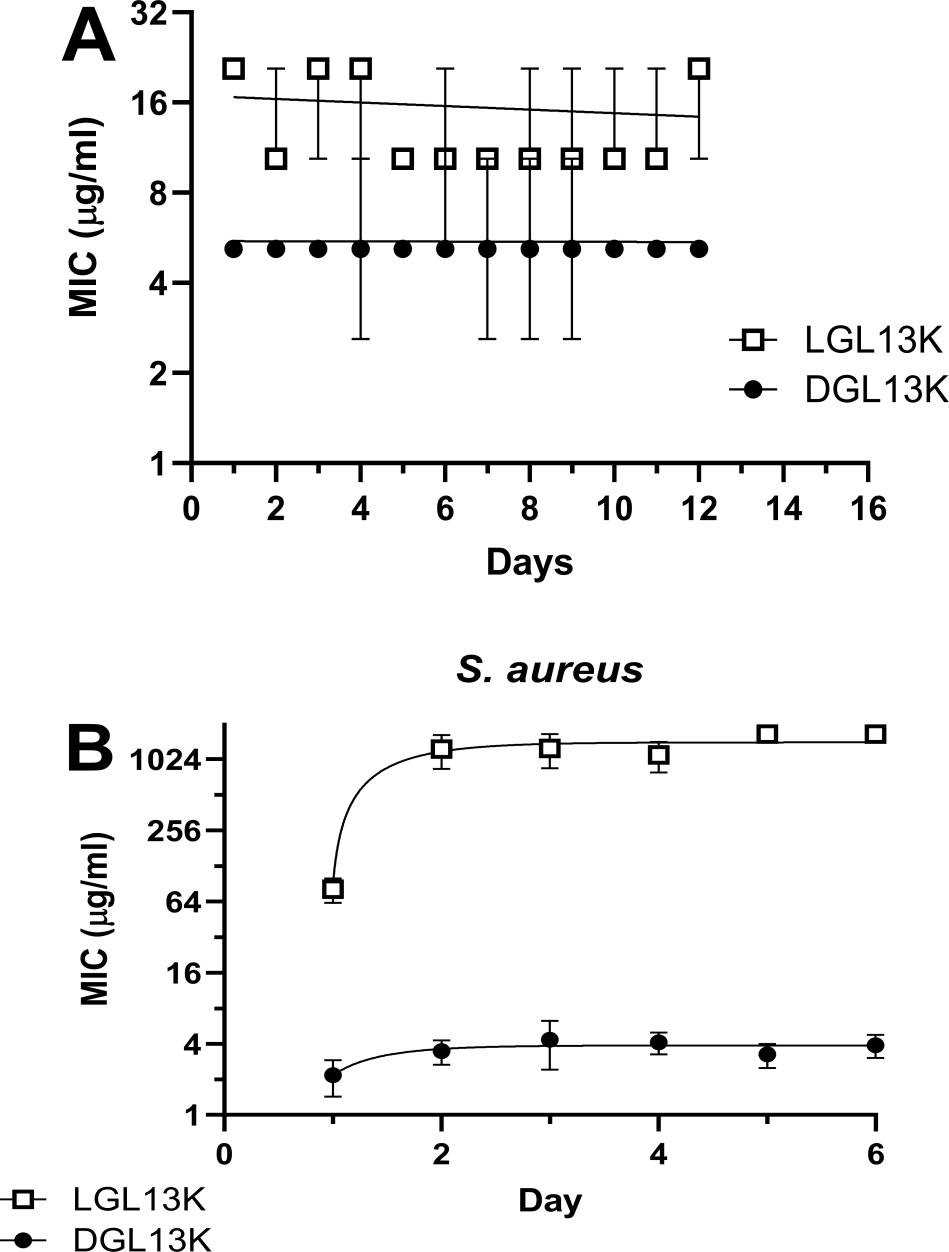
Acquired resistance of *P. aeruginosa* (**A**) and *S. aureus* (**B**) after serial MIC assays for 12 and 6 days, respectively. Bacteria were treated with LGL13K (□) or DGL13K (●) in successive MIC assays, which were each inoculated with the culture at 0.5× MIC from the previous day. Daily MICs are shown as the mean ± 95% CI of two (*P. aeruginosa*) or three (*S. aureus*) independent experiments, each performed in quadruplicate (*N* = 7–12).

The Gram-negative bacteria *P. aeruginosa* are highly susceptible to both LGL13K and DGL13K, showing only a twofold to fourfold difference in MIC between the two peptide isomers (27, 28). In fact, in a serial peptide exposure experiment, the MIC only increased about twofold for LGL13K, and this increase was not statistically significant, suggesting that the bacteria are not able to mount resistance to either peptide stereoisomer (27). To validate this finding, *P. aeruginosa* was serially exposed to LGL13K or DGL13K. As expected, neither LGL13K nor DGL13K showed an increased MIC after serial exposure for up to 12 days (Fig. 2A).

In contrast to *P. aeruginosa*, Gram-positive bacteria are relatively resistant to LGL13K, but not DGL13K (15, 28). To characterize further this stereoselective resistance, these studies were extended to the Gram-positive bacteria *S. aureus*. This species shows a lower initial MIC to LGL13K than the previously tested Gram-positive species (15, 28), which allows for further selection with LGL13K. Figure 2B shows that the MIC for LGL13K increased on day 2 of selection, reaching a 20-fold increase on day 5. In contrast, the initial MIC for DGL13K was about 40-fold lower than that for LGL13K and increased less than twofold over 6 days of selection (Fig. 2B), consistent with our results with other Gram-positive bacteria (15).

Bacteria selected with either DGL13K or LGL13K for 5–6 days were cultured in the absence of peptide, and individual colonies were isolated for further study (*peptide-selected isolates*).

### Cross-over resistance

**Fig 3:**
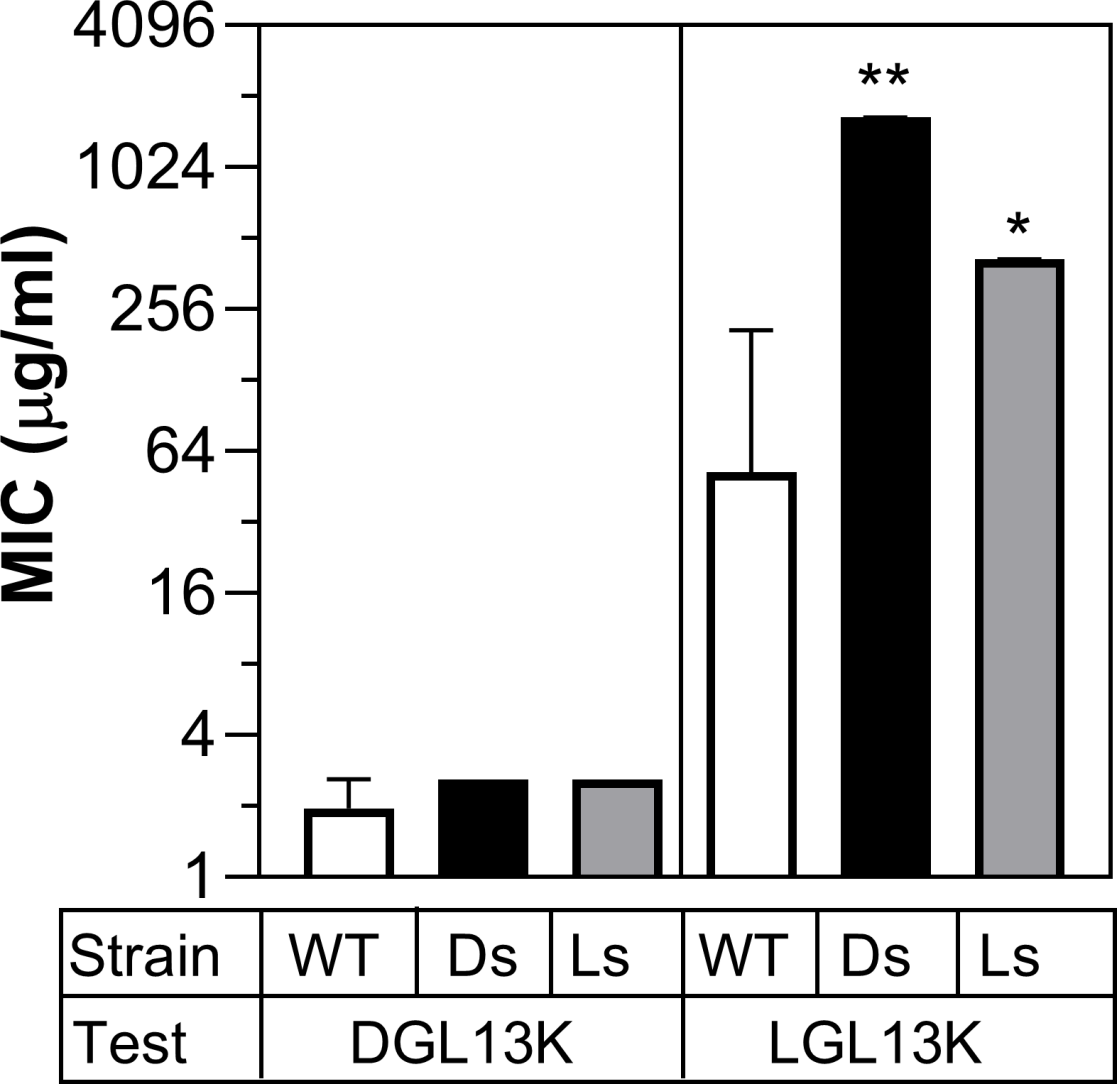
Cross-over MIC assays. Wild type (WT), DGL13K-selected (Ds), or LGL13K-selected (Ls) isolates were analyzed in MIC assays against the test peptide, DGL13K or LGL13K. The MIC was determined in 2–5 independent experiments and expressed as median MIC ± 95% CI. *, different from WT, *P* < 0.002; **, different from WT, *P* < 0.0001; *N* = 6–21.

To further explore the different levels of resistance induced by the two stereoisomers, the peptide-selected isolates were tested against both stereoisomers (Fig. 3). The DGL13K selected isolates remained susceptible to DGL13K, consistent with the selection experiments shown in Fig. 2B. Similarly, the LGL13K-selected isolates retained an increased MIC for this peptide, compared to WT bacteria (Fig. 3). In contrast, the LGL13K-selected isolates were readily killed by DGL13K, suggesting that resistance was selective for the L-isomer of this peptide. Surprisingly, the DGL13K-selected isolates became resistant to LGL13K, although they had not previously been exposed to this stereoisomer (Fig. 3). These results suggest that both peptide stereoisomers induce similar resistance mechanisms, but these mechanisms are not effective against the D-isomer, which “resists the resistance.”

### Genome sequencing

To investigate the molecular mechanism behind the increased MIC for LGL13K, DGL13K-selected and LGL13K-selected isolates of *S. aureus* were submitted for whole-genome sequencing. The analysis was focused on mutations that distinguished the peptide-selected isolates from the *S. aureus* Xen 36 wild-type genome. As shown in Table 1, four different mutations were identified in the isolates. Three of the four mutations mapped to the biosynthetic pathways for chorismate and menaquinone (KEGG: map00400 and map00130). All four DGL13K-selected isolates were mutated in *aroF* (DAHP synthase; EC 2.5.1.54; SAOUHSC_01852), which catalyzes the first step in the synthesis of chorismate (shikimate pathway) (29). LGL13K-selected isolates were mutated in *menA* (EC 2.5.1.74; SA0894) (1/1) or *menH* (EC 4.2.99.20; SAOUHSC 00984) (3*/*3), which act in the conversion of chorismate to menaquinone. The *menH* mutants (3/3) also showed mutation of *frsA* (EC 3.1.1.1; GenBank: QHL66799.1), which acts as a switch between respiration and fermentation (30). Notably, these mutations appear to be novel and were not identified in a recent screen for the AMP-induced resistome in *S. aureus* (14).

**TABLE 1:** Genome sequencing of peptide-selected isolates^*a*^

Selection	Colony	GENE	NT change	AA change
LGL13K1	A	*frsA*	A→T	Tyr256Asn
	B			
	C			
	A	*menH*	A→T	Ile106Leu
	B			
	C			
LGL13K2	D	*menA*	C→G	Ala91Pro
DGL13K1	E	*aroF*	G→A	Arg210Cys
	F			
	G			
	H			

^*a*^ Bacteria were selected with LGL13K (two selection experiments) or DGL13K (one selection experiment), and 1–4 individual colonies from each set were sequenced. The mutated genes, nucleotide (NT) change, and the corresponding location and change of protein sequence (AA) are shown.

### Characterization of small colony variants

**Fig 4:**
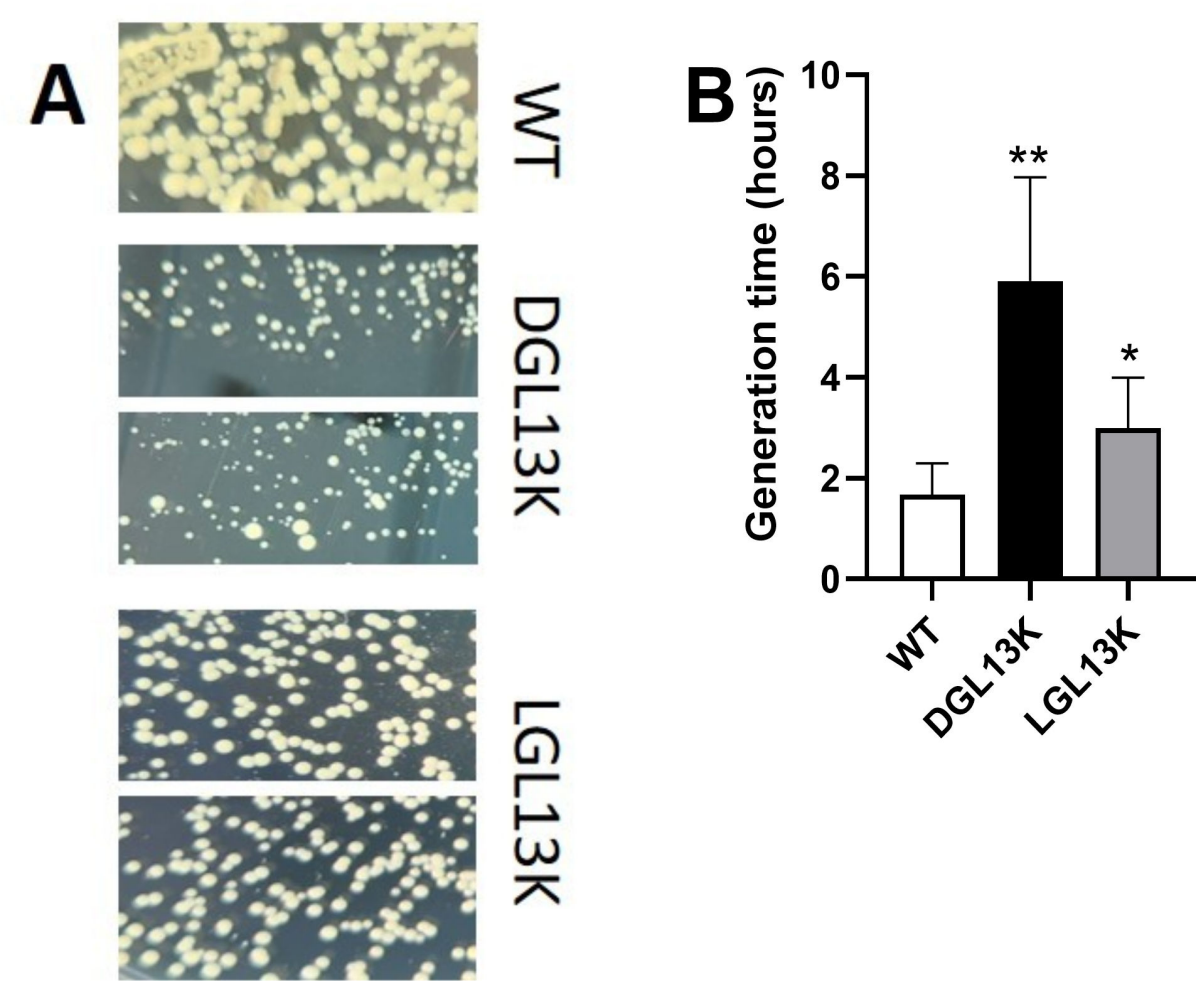
Growth characteristics of peptide-selected isolates. (A) Wild type (WT) and two isolates, each selected with DGL13K or LGL13K, were plated on THB agar and photographed to show the relative colony sizes. The images are representative of three independent cultures. (B) The generation time (doubling time) was calculated from exponential growth curves for WT, DGL13K-selected, and LGL13K-selected isolates. Data from four independent experiments with 1–2 WT samples and 3–6 isolates are shown as mean ± 95% CI, *N* = 6–21. *, different from WT, *P* < 0.05; **, different from WT, *P* < 0.001.

The peptide-selected isolates showed colony morphology reminiscent of SCVs (Fig. 4A), which are associated with antibiotic resistance (31–34). The LGL13K-selected colonies were somewhat smaller than WT colonies, while DGL13K-selected colonies were substantially smaller than WT colonies. This difference was quantified from exponential growth curves. The generation time (doubling time) for DGL13K-selected isolates was 3.5-fold longer than for wild-type cultures, while LGL13K-selected isolates showed a 1.8-fold increase in doubling time (Fig. 4B), consistent with the observed colony morphology.

**Fig 5:**
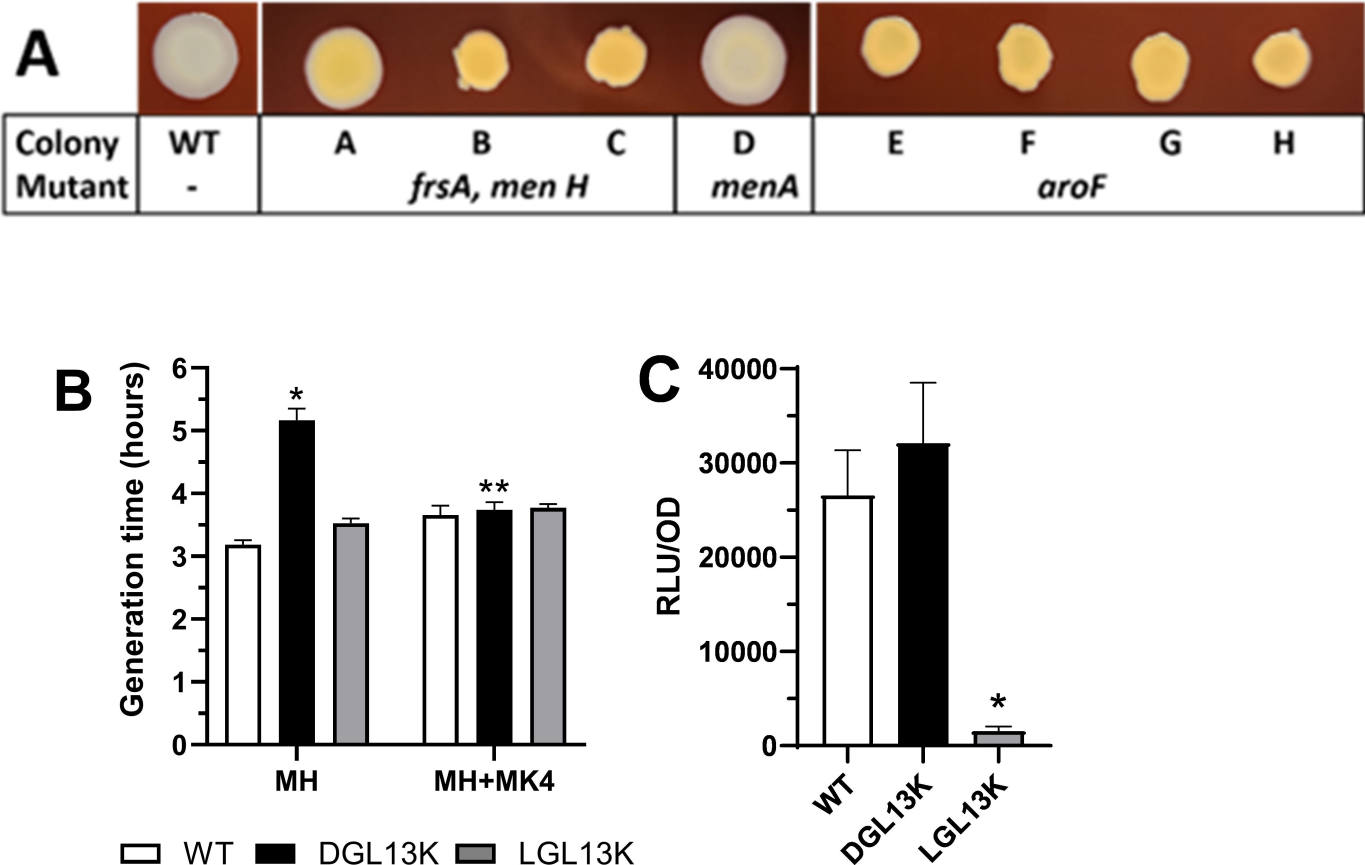
Characterization of peptide-selected isolates. (A) Aliquots of WT, DGL13K-selected, and LGL13K-selected isolates were cultured on blood agar for 2 days. Colony ID refers to Table 1. Mutant lists the genes mutated in each isolate (see Table 1). (B) Generation time (doubling time) for WT (white bar), DGL13K-selected (black bar), and LGL13K-selected (gray bar) isolates cultured in Mueller-Hinton broth with (MH+MK4) or without (MH) menaquinone supplementation. Data from two independent experiments are shown as mean ± 95% CI; *, different from MH WT and LGL13K; **, different from MH DGL13K; *P* < 0.0001, *N* = 3–7. (C) ATP activity in peptide-selected isolates. Bacterial luminescence (RLU) was recorded at the stationary phase of growth and expressed relative to the OD600 of the culture. The average OD600 in each group was WT = 0.7; DGL13K = 0.5; LGL13K = 0.6. The data from eight independent experiments are shown as mean ± 95% CI. *, different from WT and DGL13K, *P* < 0.0001, *N* = 26–35.

In addition to longer generation times and the resulting small colony size, SCVs of *S. aureus* have been characterized by a lack of pigmentation and auxotrophism caused by mutations in metabolic pathways that lead to defective electron transport and decreased ATP production (32, 33). *S. aureus* Xen36 is a derivative of ATCC 49525 and produces non-pigmented (cream colored) colonies (35). The lack of pigmentation of WT Xen36 was confirmed by culture on blood agar for 2 days (Fig. 5A). In contrast, the DGL13K-selected variants showed strong pigmentation, while the LGL13K-selected variants showed varying levels of pigmentation (Fig. 5A). Thus, higher levels of pigmentation were correlated with smaller colony size. The culture on blood agar did not affect relative colony sizes: DGL13K-selected isolates produced considerably smaller colonies than WT bacteria.

SCVs are typically auxotrophic, including menadione auxotrophy (36–38), which makes the bacteria unable to synthesize menaquinone. Given the mutations identified in the chorismate and menaquinone biosynthetic pathways (Table 1), we tested the growth rates of peptide-selected isolates cultured in the presence or absence of menaquinone. The generation times of WT and LGL13K-selected isolates were not affected by this supplementation, while the longer generation time of DGL13K-selected isolates was reduced to that of the other samples in the presence of menaquinone (Fig. 5B).

Menadione-auxotrophy is associated with electron transport deficiency that reduces ATP production (37). *S. aureus* Xen36 has been engineered to express a *Photorhabdus luminescens* luciferase (35), which can be used as an indicator of cellular ATP levels. To determine if peptide-selected isolates showed defects in ATP production, the bacteria were cultured to stationary phase, and the relative ATP-dependent luminescence was recorded. Figure 5C shows that the DGL13K-selected isolates expressed similar ATP levels as WT bacteria, while the LGL13K-selected isolates were highly deficient in ATP content. This pattern was not affected by culture in the presence of menaquinone (not shown).

**Fig 6:**
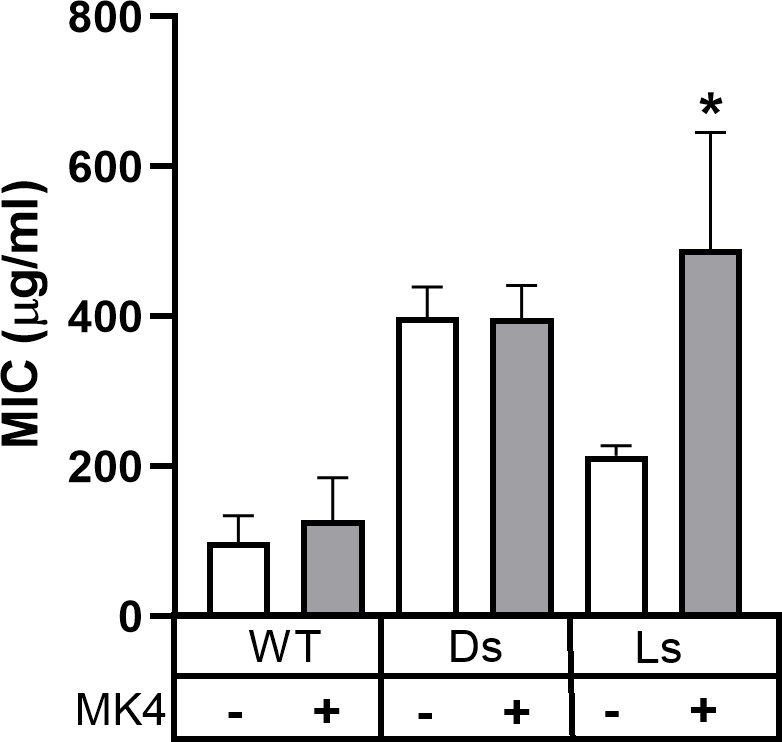
MIC of LGL13K in the presence and absence of menaquinone. *S. aureus* Xen36 (WT), DGL13K-selected isolates (Ds), and LGL13K-selected isolates (Ls) were cultured in the absence (−) or presence (+) of menaquinone (MK4). MICs (µg/mL) were determined in three independent experiments and expressed as mean ± 95% CI; *N* = 5–10. *, different from “Ls–MK4,” *P* < 0.005.

Addition of menaquinone to bacterial membranes has been linked to increased resistance to a cationic antimicrobial peptide (39). To determine how menaquinone affects resistance to LGL13K, peptide-selected isolates were cultured in the presence or absence of menaquinone (Fig. 6). WT bacteria show a low level of resistance, which was not affected by the addition of menaquinone. DGL13K-selected isolates showed a high level of resistance to LGL13K, and this was not further affected by the addition of menaquinone. In contrast, LGL13K-selected isolates showed an intermediate level of resistance, which was further increased by the addition of menaquinone (Fig. 6).

The increased pigmentation of the SCVs identified in this study (Fig. 5A) is an unusual observation since SCVs are typically defined by slow growth and lack of pigment, e.g., reference 40. On the other hand, both the SCV phenotype (31–34, 41) and increased pigmentation by staphyloxanthin (42) have been associated with antibiotic resistance. Staphyloxanthin production in *S. aureus* is regulated by the alternative transcription factor sigmaB (*sigB*) through an upstream sigmaB-dependent promoter in the staphyloxanthin biosynthetic operon (43). Deletion of *sigB* eliminates pigmentation (44). Thus, the upregulation of staphyloxanthin production in peptide-selected isolates (Fig. 5A) is consistent with increased *sigB* activity. To confirm that *sigB* activity is increased in peptide-selected isolates, additional cellular processes that are regulated by this transcription factor were analyzed.

**Fig 7:**
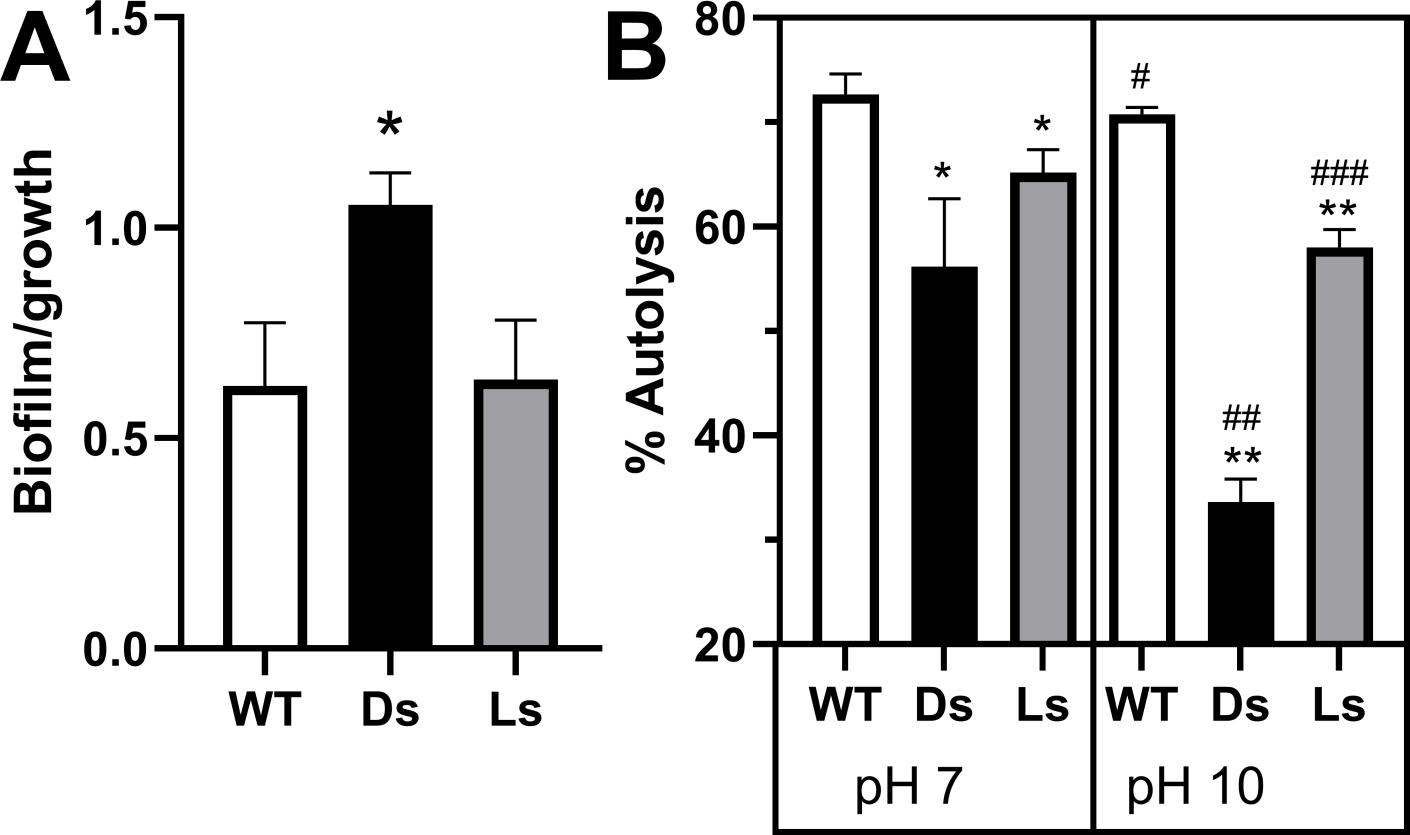
Biofilm formation (**A**) and autolysis (**B**) of peptide-selected isolates. (A) Forty-eight-hour biofilms of *S. aureus* Xen36 (WT), DGL13K-selected isolates (Ds), and LGL13K-selected isolates (Ls) were stained with crystal violet, and the OD570 of crystal violet (Biofilm) was expressed relative to culture density (Growth; OD630). Biofilm/growth is expressed as mean ± 95% CI. One to two WT, 4 Ds isolates, and 3–4 Ls isolates were tested in duplicate in three separate experiments; *N* = 12–24. The values for each experiment were normalized to the mean of the WT samples for that experiment. Data were analyzed by Brown-Forsythe and Welch analysis of variance (ANOVA) with Tukey’s multiple comparison test. *, different from WT, *P* < 0.02. (B) Autolysis of *S. aureus* Xen36 (WT), DGL13K-selected isolates (Ds), and LGL13K-selected isolates (Ls) that were pre-incubated at pH 7 (left panel) or pH 10 (right panel). % Autolysis was calculated after 2 ¼ h as described in Materials and Methods. WT, 3–4 Ds isolates, and 4 Ls isolates were tested in quadruplicate; *N* = 4–16. WT was compared to Ds and Ls for each pH by Brown-Forsyth and Welch ANOVA. *, different from WT at pH 7, *P* < 0.0004. **, different from WT at pH 10, *P* < 0.0001. WT, Ds, and Ls were independently compared at pH 7 and pH 10 by Student’s *t*-test. #, different from WT pH 7, *P* < 0.03; ##, different from Ds pH 7, *P* < 0.0001. ###, different from Ls, pH 7, *P* < 0.0001. The experiment was repeated with similar results.

Overexpression of *sigB* in SCVs has been linked to increased biofilm formation (45). DGL13K-selected SCVs showed significantly increased biofilm formation when compared to LGL13K-selected isolates and wild-type *S. aureus* (Fig. 7A), suggesting that *sigB* is upregulated in the DGL13K-selected SCVs compared to WT bacteria.

*sigB* is also an exclusive regulator of Alkaline Shock Protein 23 (Asp23) (46), a cell surface protein that is involved in autolysis (47) and the bacteria’s response to alkaline growth conditions (48). Indeed, autolysis is reduced in the D-selected and L-selected isolates compared to wild-type bacteria (Fig. 7B), suggesting that Asp23 is upregulated in the peptide-selected isolates. The role of Asp23 was further confirmed by testing the effect of alkaline shock on autolysis (Fig. 7B). WT samples showed a small decrease in autolysis after alkaline shock, while both D-selected and L-selected isolates showed larger reductions in autolysis in the face of alkaline shock at pH 10. These results are consistent with upregulation of Asp23 in peptide-selected isolates exposed to alkaline conditions.

## DISCUSSION

We have previously reported that *S. aureus* is relatively resistant to the AMP LGL13K but not the stereoisomer DGL13K (28). In this study, we sought to determine the cellular consequences of this difference. Surprisingly, both peptides selected for mutations in the chorismate/menaquinone biosynthetic pathway, resulting in increased resistance to LGL13K but not DGL13K. Thus, bacteria selected with DGL13K become resistant to the stereoisomer LGL13K, but DGL13K can evade this resistance (“*resisting the resistance*”). Cross-resistance between different AMPs has been observed experimentally (19–21). Indeed, the selection of bacteria resistant to one AMP may increase, decrease, or have no effect on the MIC of a different AMP, as reviewed in reference 9. This has led to the proposal that widespread use of AMPs could render the host-defense peptides ineffective against invading bacteria by “arming the enemy” (17, 18). However, it has been countered that the AMPs nisin and polymyxin have been in general use for decades without affecting host defenses (22). The present results suggest that resistance to an AMP can be overcome by a closely related AMP, which bodes well for future clinical use.

Unlike early suggestions that AMPs, which act on the cell membrane, are unlikely to cause extensive resistance (49, 50), the present results clearly support subsequent findings that membrane-active peptides, including LGL13K (51), can cause resistance in *S. aureus* (52–54). Our genome sequencing data suggest that the chorismate/menaquinone pathway plays a role in resistance to LGL13K. Indeed, this pathway has previously been associated with activity and resistance to AMPs (39, 55), and *menF* is part of a mutated gene complex in pexiganan-selected *S. aureus* (21). On the other hand, *men* genes were not included in the “resistome” identified upon selection of *S. aureus* with the endogenous AMP LL-37 or engineered LL-37 derivatives (14).

Both peptide isomers were selected for mutations in the chorismate/menaquinone pathway. The DGL13K-selected isolates showed a mutation in *aroF* (DAHP synthase), which catalyzes the first step in the synthesis of chorismate (shikimate pathway) (29, 56). Based on the structures of DAHP synthase in other species, the conserved Arg residue at position 210 contributes to the substrate-binding site for phosphoenolpyruvate (57). It remains to be determined if this mutation affects substrate binding.

Menaquinone is synthesized from chorismate in the vitamin K pathway, which includes the genes *menA* and *menH* that were mutated in two distinct selections with LGL13K. The mutation identified in *menH*, Ile106Leu, is conservative and not located in the catalytic triad or a conserved region of the protein (58). Thus, the functional significance of this mutation is unclear. However, these mutants also showed mutation of *frsA*, which acts as a switch between respiration and fermentation (30) and could affect ATP production. Indeed, ATP content is greatly reduced in the LGL13K-selected isolates (Fig. 5C). Overexpression of *frsA* was noted in bacteria that are resistant to the glycopeptide Phleomycin (59). FrsA has also been described in *Vibrio vulnificus* (GenBank: HM172799.1), where it increases glucose fermentation by interaction with glucose-specific enzyme IIA(Glc) under oxygen-limited conditions (60). Alignment of the FrsA protein sequences of *S. aureus* and *V. vulnificus* did not reveal any strong similarity in the sequence surrounding the mutated Tyr256 of *S. aureus* (Table 1). Thus, the functional implication of this mutation remains to be determined.

To validate the role of the menaquinone pathway in the selected mutants, the bacteria were cultured in the presence of menaquinone in two separate experiments: when the effect of menaquinone on growth rate was tested, the DGL13K-selected isolates showed faster growth, while LGL13K-selected isolates and WT bacteria were unaffected. Conversely, when MIC was determined in the presence of menaquinone, LGL13K-selected isolates showed an increase in MIC, while DGL13K-selected isolates and WT bacteria were unaffected. The DGL13K-selected isolates grow more slowly, with smaller colonies than the LG13K-selected isolates. It appears that menaquinone can overcome this deficit and return their growth rate to that of WT cells (Fig. 5B). LGL13K-selected isolates already exhibit a growth rate similar to WT cells, and this is not affected by the addition of menaquinone.

A similar pattern emerges for the effect of menaquinone on the MIC of LGL13K. DGL13K-selected isolates are already highly resistant to LGL13K, and this is not further increased by the addition of menaquinone. Conversely, the intermediate resistance of LGL13K-selected isolates can be increased by the addition of menaquinone. In contrast, the low resistance of WT bacteria is not affected by menaquinone, presumably since these bacteria already produce sufficient amounts of this lipid.

The observed differences between DGL13K and LGL13K-selected isolates extend from the effect of menaquinone to the production of the carotenoid staphyloxanthin, which lends the colonies their golden color and has been assigned a protective effect against oxidative stress (61). The golden colony color was most pronounced in the DGL13K-selected isolates, suggesting that the *aroF* mutation, in particular, affected staphyloxanthin production. Increased staphyloxanthin production is associated with increased membrane rigidity, and it has been suggested that this could enhance resistance to cationic AMPs (42). Indeed, the golden isolates showed increased resistance to LGL13K, while DGL13K was able to overcome this barrier, suggesting that the structure of the D-stereoisomer allows it to bypass the more rigid membrane structure. It has previously been reported that LGL13K and DGL13K differ in their interaction with components of the Gram-positive cell wall (15, 62). The present results suggest that this concept extends to interaction with components of the bacterial cell membrane. Experiments with model membranes have found that LGL13K forms a beta-sheet structure in the presence of negatively charged liposomes and destabilizes the membrane by removing lipid micelles (63). Harmouche et al. (51) similarly found that LGL13K transitions from random coil in solution to beta-sheet conformation in the presence of negatively charged lipid membrane, which leads to deformation of the membrane and opening of the bilayer. The ability to form beta-sheets and self-assembled nanostructures in solution is faster for DGL13K than LGL13K and is lacking in a randomized GL13K sequence. Thus, these nanostructures correlate with the antimicrobial activity of each peptide (64). Together, these results support the notion that L- and D-isomers of AMPs are not perfect mirror images but display subtle differences in secondary structure that manifest in differentiated antimicrobial activity, beyond their differences in proteolytic susceptibility (15, 65).

In addition to their golden color, the peptide-selected isolates displayed as SCVs, which have been associated with antimicrobial resistance (31, 33), including resistance to AMPs (34, 41). This colony morphology was most pronounced for the DGL13K-selected *aroF* mutants, but was still notable for the LGL13K-selected mutants. In addition to colony morphology, SCVs are frequently characterized by auxotrophism for menadione, hemin, and/or thymidine (37). Mutations in *menC*, *menD*, *menE*, or *menF* block the biosynthesis of menadione, which renders the bacteria unable to synthesize menaquinone (38). Indeed, LGL13K-selected mutants, but not DGL13K-selected mutants, showed greatly reduced ATP levels, suggesting that the former exhibit defective electron transport, which is associated with menadione auxotrophism (37).

The SCVs identified in this study differ from “conventional” SCVs in several ways (Table 2). SCVs typically present as small colonies with defects in electron transport that lead to reduced electrochemical gradient, resistance to cationic antibiotics—including AMPs, decreased ATP levels and cellular growth, and *decreased* pigment formation (37). The DGL13K-selected isolates present with a small colony morphology and resistance to LGL13K but not DGL13K. The ATP content is not affected, but the colonies show increased pigment formation. LGL13K-selected isolates show moderately reduced colony size and greatly reduced ATP content. However, the resulting colonies only display a moderate increase in pigment formation.

**TABLE 2:** Properties of small colony variants selected with DGL13K or LGL13K

	Conventional SCV	DGL13K-selected	LGL13K-selected
Colony size	Small	Small	Intermediate
Generation time	Slow	Slow (3.5 × WT)	Decreased (1.8 × WT)
Resistance	Cationic AMP resistance	LGL13K, increased DGL13K, no effect	LGL13K, increased DGL13K, no effect
Mutated genes	Multiple	*aroF*	*menA, menH, frsA*
Pathway	Multiple	Shikimate	Menaquinone
Colony color	Cream/white	Golden	Some golden
Auxotrophism	Menadione, hemin, and/or thymidine	Menaquinone	Not detected
ATP production	Low	Normal	Low
Effect of menaquinone on resistance (MIC)	Not tested for DGL13K/LGL13K	Highly resistant w/o MK4 No effect of MK4	Medium resistance w/o MK4 Increased resistance with MK4

The increased coloration of the SCVs described in this report suggests that additional regulatory changes have taken place. Staphyloxanthin production is regulated by the alternative sigma factor sigmaB, and deletion of *sigB* is associated with loss of staphyloxanthin production (44, 66, 67). Indeed, *sigB* is a regulator of multiple virulence genes and stress responses (67, 68). To determine if the increased coloration of the peptide-selected isolates was associated with increased *sigB* activity, additional processes regulated by *sigB* were tested, including biofilm formation (45), autolysis, and alkaline shock (47, 48). The latter two processes are mediated by Alkaline Shock Protein 23 (Asp23), a cell surface protein that is exclusively regulated by *sigB* (46, 47). Increased biofilm formation and decreased autolysis, which was further reduced by alkaline shock, all support that Asp23 and *sigB* expression are increased in the peptide-selected isolates. The effects were most pronounced in the DGL13K-selected isolates, consistent with the deeper color produced by these isolates.

The mechanisms behind the differential effect of DGL13K and LGL13K on *S. aureus* resistance remain to be elucidated. The finding that DGL13K induced resistance to the stereoisomer LGL13K but not to DGL13K itself suggests that peptide primary structure is responsible for inducing bacterial defense mechanisms, but the peptide secondary structure determines if the defense mechanisms are effective against each peptide. In this context, cationic peptides may be recognized as alkaline mediators that trigger an Asp23 response (48). Introduction of cellular stress by sub-MIC concentrations of vancomycin has similarly been reported to increase *sigb* and *asp23* expression in vancomycin-resistant *S. aureus* (69).

It is notable that the stress response recorded for DGL13K-selected isolates is not sufficient to increase resistance to this peptide. We have now attempted to develop resistance to DGL13K in *S. aureus* (this report), *Enterococcus faecalis* (15), *Streptococcus gordonii* (15), *Pseudomonas aeruginosa* (this report [27]), and *Porphyromonas gingivalis* (70) and failed in each species. Notably, *S. aureus* and *P. gingivalis* showed mutations associated with bacterial resistance, but neither species was able to mount resistance to DGL13K, although *P. gingivalis* showed increased tolerance to the peptide (70). Thus, while it may not be possible to avoid an antimicrobial resistance/stress response in bacteria exposed to novel antibiotics, it appears possible to design antibiotics that can overcome this response by “resisting the resistance.”
