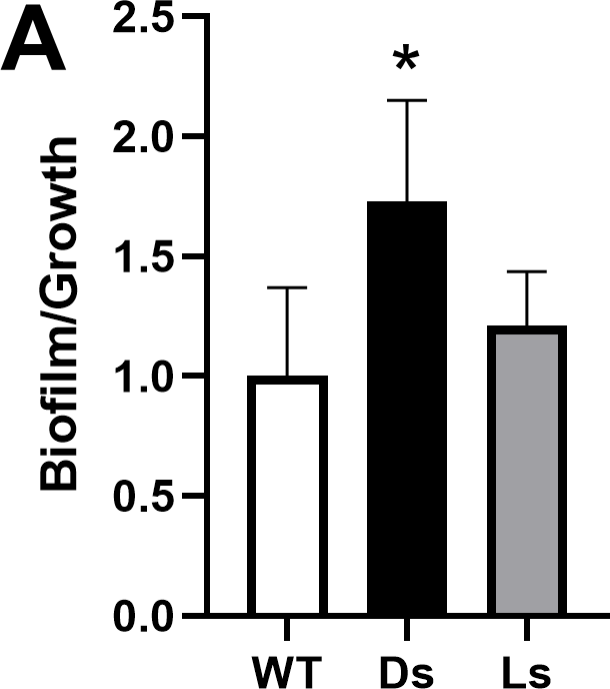# Erratum for Gorr, “Resisting the resistance: the antimicrobial peptide DGL13K selects for small colony variants of *Staphylococcus aureus* that show increased resistance to its stereoisomer LGL13K, but not to DGL13K”

**DOI:** 10.1128/jb.00529-25

**Published:** 2026-02-17

**Authors:** Sven-Ulrik Gorr

## ERRATUM

Volume 207, no. 6, e00505-24, 2025, https://doi.org/10.1128/jb.00505-24. Figure 7A should appear as shown in this erratum. Regrettably, the figure originally published was an earlier version and did not represent the final complete data. The figure legend is correct, and the results and conclusions drawn remain unchanged. I apologize for this error.

**Fig 7:**